# Draw-spun, photonically annealed Ag fibers as alternative electrodes for flexible CIGS solar cells

**DOI:** 10.1080/14686996.2018.1552480

**Published:** 2018-11-30

**Authors:** Yujing Liu, Simon Zeder, Sen Lin, Romain Carron, Günter Grossmann, Sami Bolat, Shiro Nishiwaki, Frank Clemens, Thomas Graule, Ayodhya N. Tiwari, Hui Wu, Yaroslav E. Romanyuk

**Affiliations:** a Laboratory for Thin films and Photovoltaics, Empa-Swiss Federal Laboratories for Materials Science and Technology, Dübendorf, Switzerland; b Department of Materials Science & Engineering, Tsinghua University, Beijing, China; c Laboratory for Transport at Nanoscale Interfaces, Empa-Swiss Federal Laboratories for Materials Science and Technology, Dübendorf, Switzerland; d Laboratory for High Performance Ceramics, Empa-Swiss Federal Laboratories for Materials Science and Technology, Dübendorf, Switzerland

**Keywords:** Ag network, CIGS solar cell, transparent conductive electrode (TCE), J-V curve, EQE, tensile test, 40 Optical, magnetic and electronic device materials: 209 Solar cell / Photovoltaics, 106 Metallic materials, 201 Electronics / Semiconductor / TCOs

## Abstract

We explore the feasibility of Ag fiber meshes as electron transport layer for high-efficiency flexible Cu(In,Ga)Se_2_ (CIGS) solar cells. Woven meshes of Ag fibers after UV illumination and millisecond flash-lamp treatment results in a sheet resistance of 17 Ω/sq and a visible transmittance above 85%. Conductive Ag meshes are integrated into flexible CIGS cells as transparent conductive electrode (TCE) alone or together with layers of Al-doped ZnO (AZO) with various thickness of 0…900 nm. The Ag mesh alone is not able to function as a current collector. If used together with a thin AZO layer (50 nm), the Ag mesh markedly improves the fill factor and cell efficiency, in spite of the adverse mesh shadowing. When Ag mesh is combined with thicker (200 nm or 900 nm) AZO layers, no improvements in photovoltaic parameters are obtained. When comparing a hybrid TCE consisting of 50 nm AZO and Ag fiber mesh with a thick 900 nm reference AZO device, an improved charge carrier collection in the near-infrared range is observed. Regardless of the AZO thickness, the presence of Ag mesh slows down cell degradation upon mechanical tensile stress, which could be interesting for implementation into flexible thin film CIGS modules.

## Graphical Abstract

**Figure:**
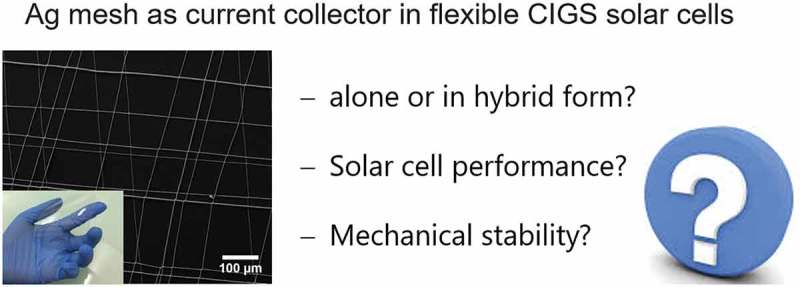

## 1. Introduction

Ag networks composed of one-dimensional nanowires and nano/microfibers have been considered as promising candidates for transparent conductive electrodes (TCEs), which are required as indispensable components in numerous optoelectronic devices such as touch panel displays, OLEDs, solar cells and smart windows [1,2]. Numerous reports on Ag networks and their applications are stimulated by advantageous characteristics of Ag networks such as (i) a low sheet resistance of 10 Ω/sq combined with a high optical transmittance above 80%, (ii) mechanical flexibility, (iii) nonvacuum deposition at low temperature that could be eventually implemented in a roll-to-roll manufacturing, and (iv) potentially lower cost as compared to widely used transparent conducting oxide (TCO) such as sputtered indium tin oxide (ITO). Consequently, Ag networks are often considered and investigated as alternative transparent electrodes for thin-film solar cells, such as those based on Cu(In,Ga)Se_2_ (CIGS). The CIGS solar cells exhibit a power conversion efficiency of up to 22.9% [3], which is currently the highest among all thin-film technologies, and can also be deposited on flexible polymer substrates [4].

The implementation of Ag networks into CIGS solar cells have been reported in several publications [5–11], attempting to replace a reference electrode consisting of a sputtered Al-doped ZnO (AZO) or ITO layer. The Ag network alone cannot function as an efficient electrode [6] and therefore, Ag networks are typically combined with other materials in hybrid structures, such as in a sandwiched structure between two TCO layers, or covered with an additional layer of metal oxides or polymers to enhance interface adhesion and prevent current leakage. For example, Kim et al. [7] fabricated Ag network-based hybrid TCEs for CIGS solar cells by sandwiching a layer of Ag nanowire between ZnO and AZO layers to restore the loose contact between Ag nanowire and the CdS buffer layer thus enhancing the lateral conduction of hybrid TCEs; the fabricated CIGS cells showed an efficiency of 11.03%, while the reference cell with sputtered ITO had an efficiency of 10.91%. In Shin’s et al. work [8], a 10-nm-thick PEDOT:PS layer was spin coated on top of Ag nanowires to form a hybrid TCE for CIGS cell, where PEDOT:PSS functioned as a filler of empty space of an electrostatically sprayed Ag nanowire network. Singh et al. [11] and Wang et al. [10] combined sputtered ZnO or nonvacuum-processed AZO with Ag nanowire to improve the adherence of Ag network with the underlying intrinsic ZnO layer, achieving up to 14%-efficient CIGS solar cells on glass substrates [10].

Mechanical flexibility appears to be one of the main advantages of Ag networks as compared to reference TCO electrodes, yet only one study implemented the Ag network into flexible CIGS cells [9]. The Ag nanowire sandwiched between two sputtered AZO layers improved durability of the CIGS solar cells by maintaining 95% of their initial efficiency after 1000 bending cycles. In comparison, devices fabricated using AZO and ITO electrodes were able to maintain only 57% and 5%, respectively, due to crack formation and delamination of the films. The solar cell efficiency was limited, however, to 6%.

In this study we test rigorously the usefulness of the Ag networks as TCE for high-efficiency (i.e. >15%) flexible CIGS solar cells. The main difference to the previous works is that the Ag network is not over-coated with another TCO or metal oxide. Instead, conductive Ag networks are fabricated by draw-spinning on a polyethylene terephthalate (PET) substrate and then laminated onto flexible CIGS solar cells that are terminated with either intrinsic ZnO or AZO. This approach is more straightforward from the technical point of view as it isolates the fabrication of the Ag mesh and the CIGS cell. A series of experiments with different thickness of AZO are conducted in order to answer the following questions:

- Is the conductive Ag mesh alone able to replace the reference AZO electrode?
- In the case of hybrid TCEs composed of Ag mesh and AZO, can the presence of Ag mesh improve any of the photovoltaic parameters as compared to reference AZO electrodes of various thicknesses?
- Can the presence of the Ag mesh slow down the degradation of solar cells subjected to bending or tensile stresses?

## 2. Experimental part

### 2.1 Fabrication of Ag mesh on PET substrates

Ag meshes were fabricated by a draw-spinning method followed with a room-temperature UV-illumination for 3 h as detailed in Ref [12,13]. AgNO_3_ was taken as precursor, polyvinylpyrrolidone (PVP, molecular weight 1,300,000) as a polymer additive and acetonitrile as solvent with weight ratio among AgNO_3_, PVP and acetonitrile as 0.4:0.06:0.54. The mixture was kept in the dark and stirred for a couple of hours until the complete dissolution of AgNO_3_ in acetonitrile. Ag fibers were draw-spun onto a PET substrates coated with ethylene-vinyl acetate (EVA) adhesive using a 60 µm needle at a speed of 0.47 m/s. Room-temperature illumination with UV-lamp for 3 h (peak wavelength 365 nm, power 250 W) was applied to reduce the AgNO_3_ precursor into metallic phase and form junctions between crossing fibers, and turn the draw-spun aligned Ag fibers into well-woven meshes. UV-lamp treated Ag mesh on PET substrates showed a sheet resistance of approximately 35 Ω/sq. In this work, the sheet resistance of Ag mesh was further decreased by an ultrashort xenon flash lamp annealing step using PulseForge1300 system from Novacentrix. The total pulse energy density was 3.48 J/cm^2^ for a pulse duration of 1.6 ms.

### 2.2 CIGS solar cell fabrication

**Figure 1.**
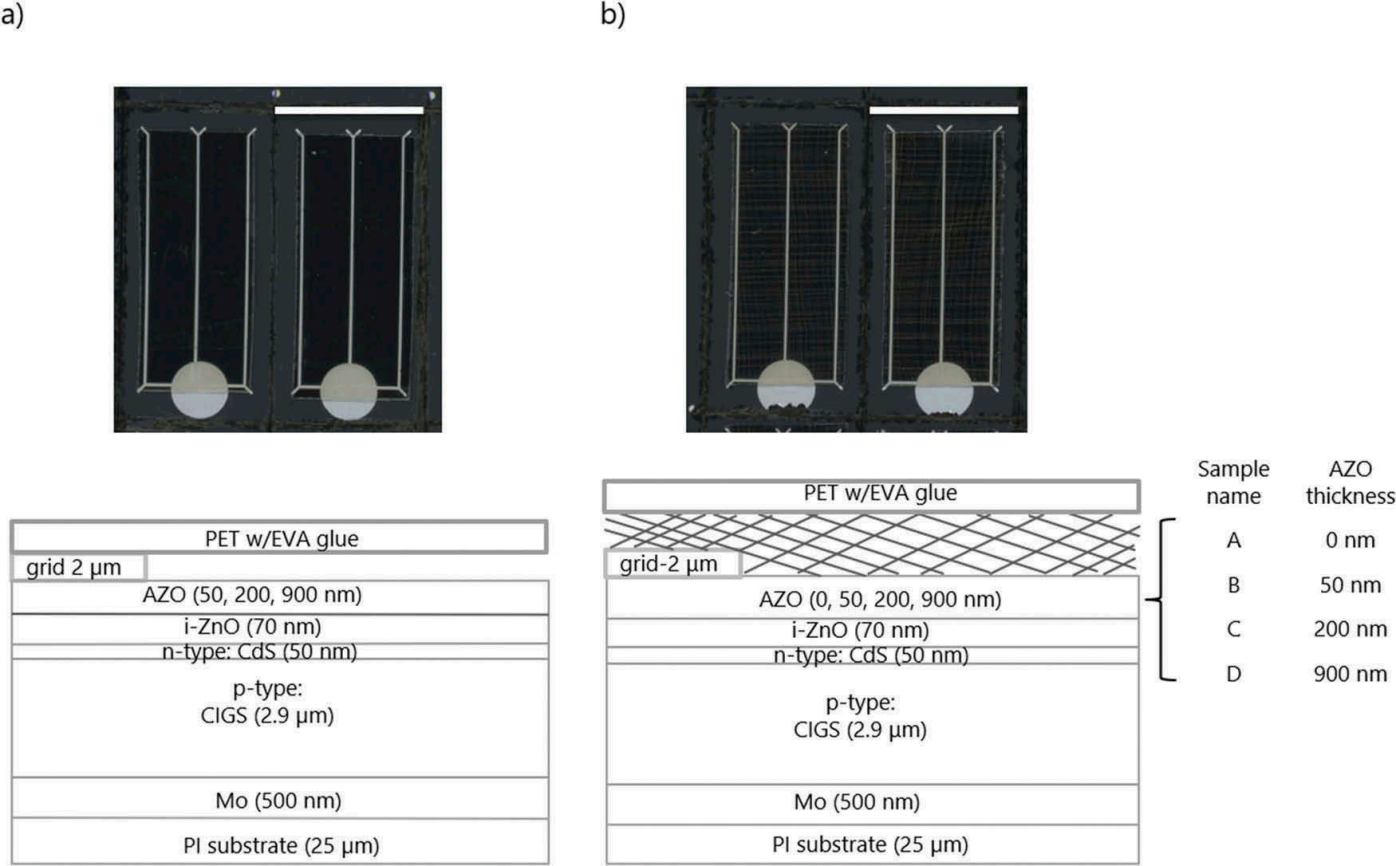
Photographs and schematic cross-sections of samples with and without integration of Ag mesh in CIGS solar cells. (a) Reference: TCE without Ag mesh, and (b) TCE with Ag mesh sample: A-0 (without AZO), B-50, C-200, and D-900. The scale bars in photograph (a) and (b) indicate 5 mm.

The layout for flexible CIGS solar cells was shown in Figure 1. Polyimide with a thickness of 25 µm (PI, UPILEX-25S) was selected as the substrate. On top of the substrate, as back contact, a layer of 500 nm Mo was prepared via dc magnetron sputtering from metallic target. CIGS absorber layer was fabricated via a three stage coevaporation procedure, as described in details elsewhere [4,14] and the thickness was ca. 2.9 µm. The n-type buffer layer CdS with a thickness of 50 nm was grown via chemical bath deposition in a basic solution containing cadmium acetate (2.3 mM), thiourea (22 mM) and ammonium hydroxide (2 M [NH3]) at 70 °C. A 70 nm layer of intrinsic ZnO (i-ZnO) was deposited with rf-sputtering. According to the cell design, the conductive AZO layer was deposited on top of i-ZnO with rf-sputtering from doped a ZnO target doped with 2 wt% of Al_2_O_3_. Three different thicknesses of AZO were considered: (i) 50 nm that does not add possess enough lateral conductivity as compared to the Ag mesh but still warrants an Ohmic contact to Ag, (ii) 200 nm that a standard thickness for the reference device empirically optimized for the highest device efficiency, and (iii) 900 nm that has a sheet resistance of ca. 10 Ohm/sq that is appropriate for larger-area CIGS modules without additional grids. The devices were completed with e-beam evaporated metal grids of 50 nm Ni followed by 2 µm Al in order to warranty identical contacting conditions for all samples. The solar cells were defined by mechanical scribing with an area around 0.55 cm^2^.

Based on the selected standard cell layout and our proposed ideas, TCE layers with and without Ag mesh were integrated into solar cells as listed in Figure 1. Cells without Ag mesh were taken as references and named according to the AZO layer thickness as Ref-50, Ref-200, and Ref-900. The cells with Ag mesh were named as A-0 (without AZO layer), B-50, C-200, and D-900. In order to compensate for the presence of the PET/EVA substrate on sample with hybrid TCE, an identical PET/EVA substrate was attached on the reference cells for the direct comparison of cells current densities and external quantum efficiency (EQE) spectra. Pictures of typical samples are shown in Figure 1. A large contact pad design was used to ensure suitable cell contacting and electrical connection to the Ag mesh.

### 2.3 Characterization

Sheet resistance of the Ag mesh on PET substrates was measured by a 4-probe contact technique in the van der Pauw configuration. Four contacts were connected with Ag paste at the corners of a 1 × 1 cm^2^ square-shaped test area. Resistivity and sheet resistance were calculated by taking the length between every two contacts into calculation. Transmittance of the Ag mesh on PET substrate as well as the PET substrate reference was measured with a UV-Vis spectrometer (UV-3600, Shimadzu, Japan) from 200 nm to 800 nm. Scanning electron microscopy (SEM) images were taken with NANOSEM 230 FEI microscope after coating the sample with a thin Pt layer.

The current–voltage curves of the solar cells were measured using a Keithley 2,400 source meter under stimulated AM1.5G illumination with intensity calibrated using a certified single crystalline silicon solar cell. The measurements were performed in forward direction (from −0.7 V to 0.8 V). The EQE of the devices was measured with a lock-in amplifier under constant light bias. The probing beam was generated by a chopped white source (900 W halogen lamp, 260 Hz) and a grating monochromator. The beam size was adjusted to ensure that the illumination area was fully inside a cell area covered by the PET foil. A certified single crystalline silicon solar cell was used as reference cell. Electroluminescence (EL) measurements were performed by imposing a 0.65 V bias voltage to the cells with Keithley 2450 source meter and the detection was taken using a digital camera ORCA-ER (C4742-80, Hamamatsu, Japan).

The tensile tests were performed in a Zwick-Roell machine (Z020, Zwick-Roell, Germany). Aluminum stripes were glued on each side of the specimens with Araldite epoxy in order to introduce a homogeneously distributed stress within the sample. The surface of the cells was left clean and not contaminated with glue. The force was applied with the supporting plates fixed in the clamps of the tensile testing machine thus preventing the damage of the cells in the zone where they have been mechanically fixed. Based on the distance between the two Al plates and the displacement of the crosshead, the strain in the cells was calculated:

$$Strain\;ratio=\frac{Displacement}{\begin{array}{c} \;\;\;\;\;\;length\;of\;sample\;\\ \;\;\;\;\left(distance\;between\;two\;Al\;plates\right) \end{array}}*100\%$$

The strain was applied at a speed of 300 µm/min to reach the required elongation. A preload of 5 N was applied before the tensile test. Multiple cycling tests (50 cycles, duration of ca. 4 h) with a strain ratio of 2.0% were carried out on cells with AZO thickness of 50 nm and 900 nm.

## 3. Results and discussion

### 3.1 Flash lamp annealing of draw-spun Ag fibers

**Figure 2.**
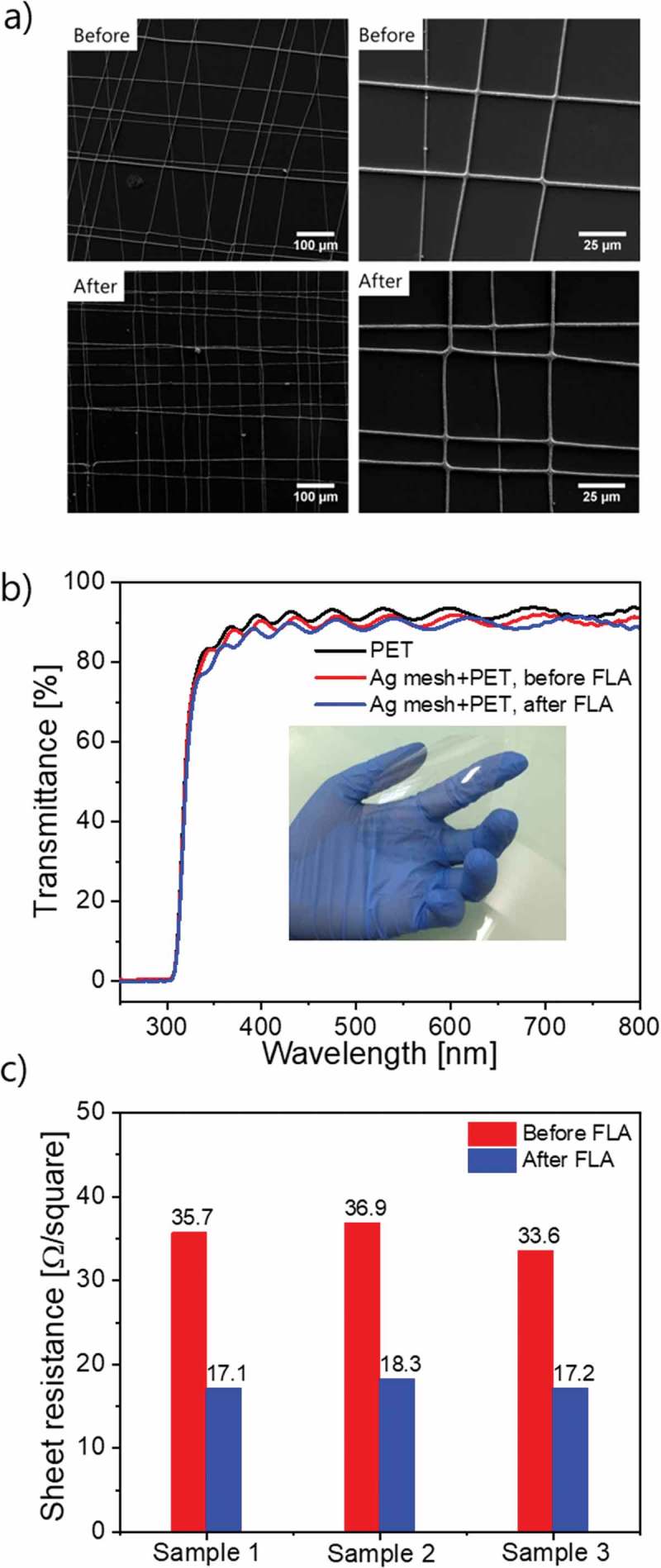
Ag mesh on PET substrates before and after flash lamp annealing: (a) SEM; (b) Optical transmittance; (c) sheet resistance.

The draw-spun Ag fibers have a diameter of ca. 2–3 μm and form a mesh with line separation of around 25 μm. Measured sheet resistance of Ag mesh on PET substrates is 35 Ω/sq and optical transmittance is above 85% for the complete stack. The presence of the Ag mesh decreases the optical transmittance of the PET foil by about 3%. The sheet resistance could be decreased from ca. 35 Ω/sq to 17 Ω/sq upon millisecond flash lamp annealing at ambient atmosphere as show in Figure 2(c)). Flash lamp annealed Ag mesh does not exhibit any changes in microstructure, as seen from SEM images in Figure 2(a). The combination of sheet resistance below 20 Ω/sq and optical transmittance above 85% in the visible light range makes Ag mesh a suitable candidate as TCE for solar cells.

### 3.2 Implementation of Ag mesh electrodes in CIGS solar cells

#### 3.2.1 Can Ag mesh alone function as TCE?

**Figure 3.**
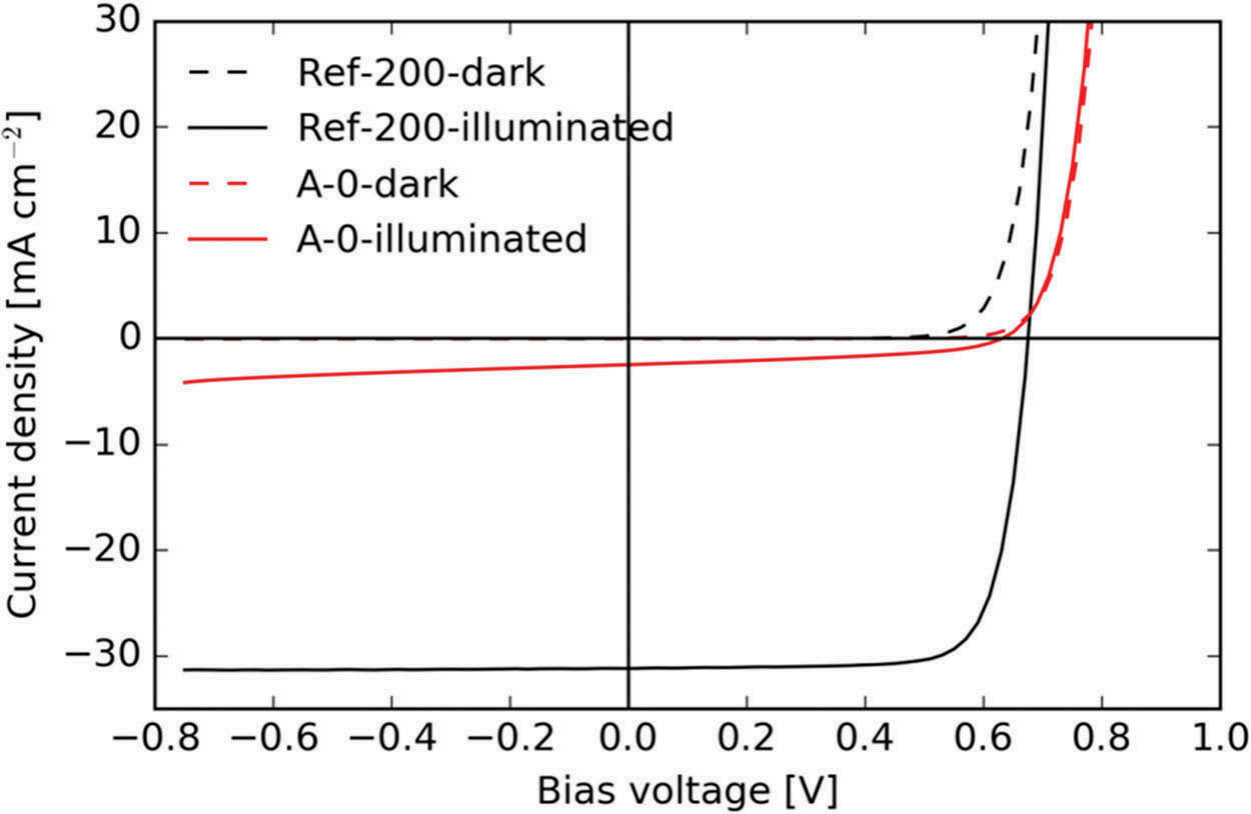
Illuminated and dark J-V curves for Ref-200 and A-0.

To address this question, CIGS cells with only a layer of Ag mesh as TCE were fabricated by attaching the Ag mesh directly on top of i-ZnO (sample A-0), and the cell performance was compared to a reference cell with AZO 200 nm (sample Ref-200). As can be seen in Figure 3, the Ag mesh as TCE only collects a small fraction of generated charge carriers, 2.2 mA/cm^2^ vs 30.9 mA/cm^2^ of Ref-200 (Table 1), resulting in a low efficiency of 0.6%. This observation confirms previous reports for Ag nanowires as TCE, which generally resulted in a very low short-circuit current density J_sc_ and hence, a poor device performance [5,6,8,15,16].

**Table 1.** Parameters of CIGS cell performance with Ag mesh against AZO 200 nm as TCE (median value of 6 cells). R_s_ and R_p_ are extracted from fits to a single diode model with 2 resistors. The J_sc_ and Eff. refer to the full cell area including the large contact pad. FF stands for fill factor and V_oc_ for open-circuit voltage.

Sample name		J_sc_ [mA/cm^2^]	V_oc_ [mV]	FF [%]	Eff. [%]	R_s_ [Ω cm^2^]	R_p_ [Ω cm^2^]
Ag vs. AZO	Ref-200	30.9	672	75.8	15.8	0.16	1924
	A-0	2.2	629	46.0	0.6	0.11	713

The low short circuit current J_SC_ for sample A-0 can be explained by the fact that most photo-generated carriers do not reach point contacts to Ag fiber mesh since the mesh spacing of 25 μm exceeds far the carrier diffusion length in CIGS. The series and parallel resistance values R_S_ and R_p_ extracted from the simple single-diode model are close to the reference device, indicating that the Ag mesh forms a sufficient electrical contact to the underlying cell structure. The cell current density remains, however, very low because charges are collected only from the limited area close to the Ag mesh contacts whereas for calculating the current density the total cell area of ca. 0.55 cm^2^ is considered.

#### 3.2.2 Ag mesh combined with AZO for hybrid TCEs

**Figure 4.**
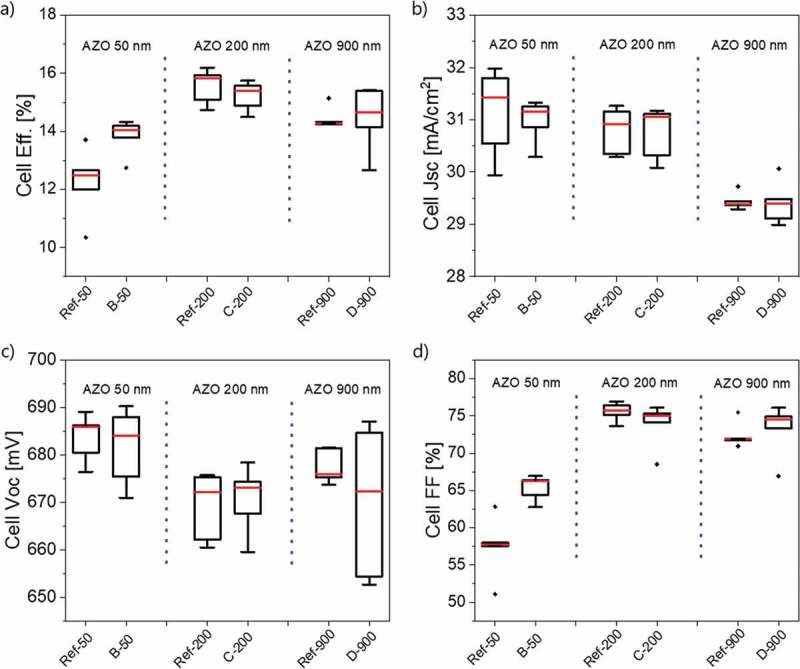
Statistical plots of performance parameters for CIGS cells with and without Ag meshes as hybrid TCE: (a) Eff.; (b) J_sc_; (c) V_oc_; and (d) FF. The J_sc_ and efficiencies are decrease by the large contact pad area. Minimum and maximum values, interquartile range as well as median value (red dash) are calculated from five to six solar cells on each sample.

The performance of CIGS solar cells with different thicknesses of AZO layers, with or without Ag meshes to form hybrid TCEs, are compared in Figure 4. Three different thicknesses of sputtered AZO, 50 nm, 200 nm, and 900 nm, are considered and compared to a reference device with 200 nm of AZO yielding the efficiency in the 15–16% range.

When AZO thickness is as low as 50 nm, a positive effect of the Ag mesh is evidenced by the improvement in fill factor (Figure 4(d)). The 50 nm AZO layer is purposely not conductive enough, resulting in inhomogeneous resistive losses and in a decreased fill factor. Adding the Ag mesh on top of the thin AZO improves the fill factor from 59% to 66%, which contributes to an increase of cell efficiency from 12.5% to 14.1%. As for cells with AZO thickness of 200 nm and 900 nm, cell fill factors are rather high as 70–75%, and the effect of Ag mesh is not as obvious as cells with AZO 50 nm.

For the AZO thickness of 200 nm no improvements of the photovoltaic parameters are detected in the range of statistical deviations. The median value for efficiency seems to be lowered because of the lower FF.

**Figure 5.**
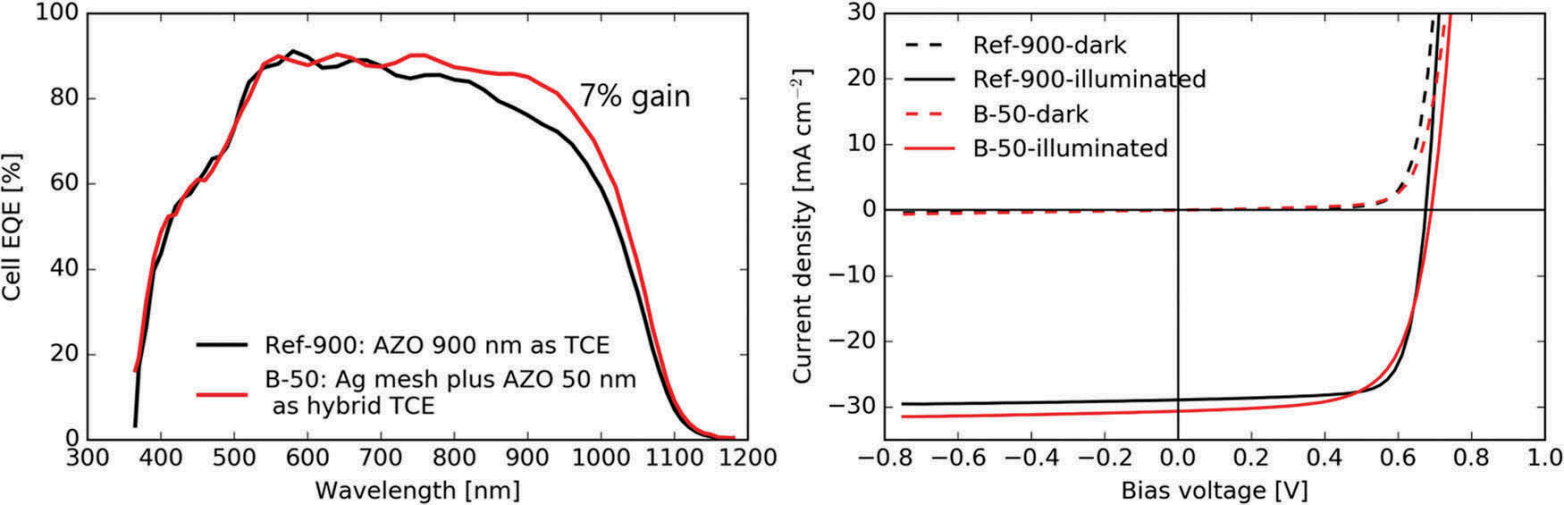
Comparison of hybrid TCEs with thin AZO of 50 nm and Ag mesh (B-50) vs. thick AZO layer of 900 nm (Ref-900): (a) EQE spectrum; (b) JV curves.

The thick AZO layer of 900 nm is relevant for industrial applications because a low sheet resistance on the order of 10 Ω/sq is required for mitigating resistive losses in large-area PV modules. However, a thick AZO layer also induces a significant parasitic absorption in the near-infrared range because of the free carrier absorption. An alternative strategy is the use of hybrid TCE composed of a thin AZO layer for local current collection completed with an Ag mesh for long-range transport. In Figure 5 the hybrid TCE sample B-50 (50 nm AZO and Ag mesh) is compared to the Ref-900 reference (900 nm AZO). The J_sc_ computed by integration of the EQE spectrum increases by approximately 7% for the hybrid TCE as compared to the thick AZO layer. A comparable change is observed in J-V measurements (31.4 mA/cm^2^ of B-50 vs. 29.4 mA/cm^2^ of Ref-900). This indicates that hybrid TCE can advantageously increase the current density by reducing the optical light absorption. In our work, however, the cell efficiency is still higher for the reference sample due to a lower fill factor of the B-50 cells. An optimized design with a mesh coupled with a relatively thicker AZO layer (e.g. 80–100 nm thick) could be considered for gaining in current while maintaining a high fill factor.

### 3.3 Device stability under tensile stress

**Figure 6.**
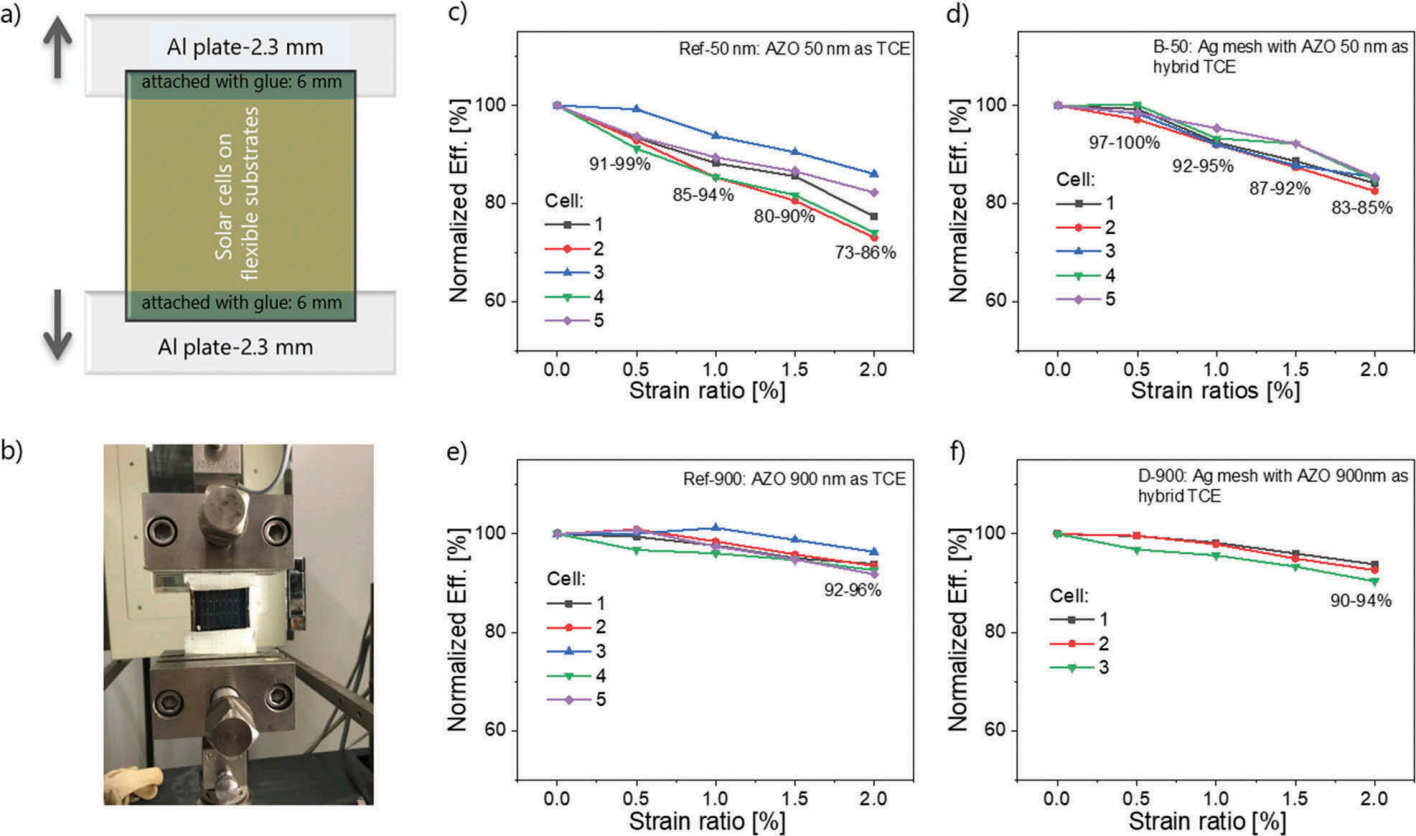
(a) Sample lay-out for tensile test; (b) Zwick-Roell test setup for tensile tests; normalized efficiency degradation under step-wise increased strain ratios from 0.0% to 2.0% (five cycles): (c) Ref-50; (d) B-50; (e) Ref-900; and (f) D-900.

Instead of bending tests, which strongly depend on the radius of curvature and allow stressing of only several cells in the middle of the substrate, the device stability in this study was assessed by tensile stretching tests. These allow homogeneous application of a shear force over a 5 × 5 cm^2^ sample. As illustrated in Figure 6(a) the samples were attached from both sides to aluminum plates with epoxy glue, and the plates were clamped in the testing setup. The solar cells were therefore kept away from force-loading area and homogeneous strain could be applied. The stress-strain tests were conducted by applying an increasing force to achieve the desired strain ratios, for example, 0.5% to 2%. Figure 6(c–f) show the cell degradation behavior with increasing strain ratios, with performing five stretching cycles at each strain ratio.

By increasing the strain ratio from 0% to 2.0%, the cell degradation behavior at 5 cycles appears relates to the thickness of the AZO layer (Figure 6). With AZO 50 nm (Ref-50 and B-50), the cells show a large efficiency degradation and the degradation rate is slowed down by the integration of an Ag mesh. Cells with 900 nm AZO thicknesses (Ref-900 and D-900) show a comparatively better stability than those with 50 nm and the effect of the Ag mesh is less pronounced. Reasons why a thinner 50 nm AZO layer is more prone to degradation as compared to thick 900 nm AZO are not clear since one would expect that a thicker AZO layer should be more brittle. One possible explanation is that even if the thick AZO layer should be more brittle (i.e. cracks are formed easier), the 50-nm AZO is so thin that even a few initial cracks are able to deteriorate rapidly its conductivity.

**Figure 7.**
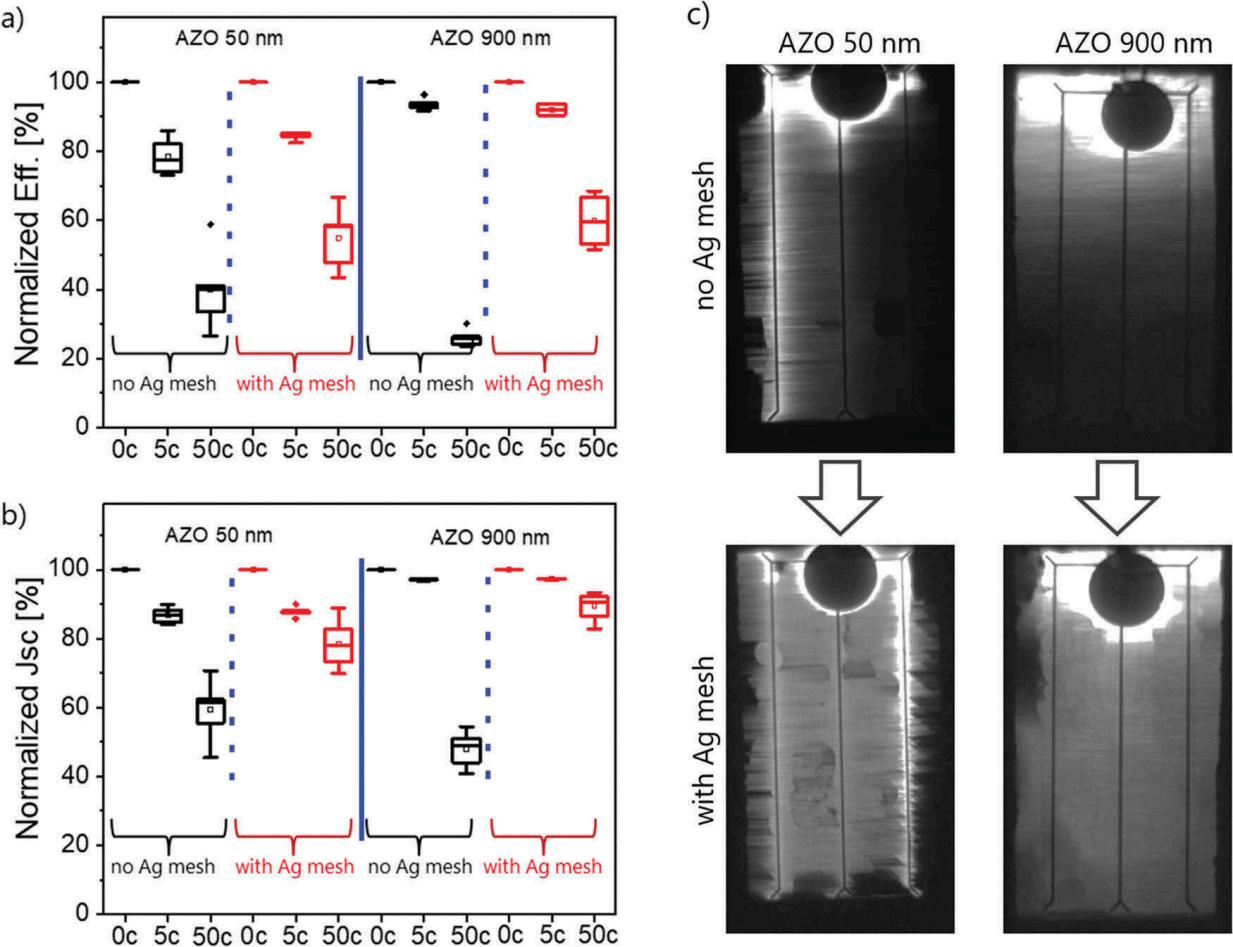
Normalized performance parameters for cells with 50 nm and 900 nm of AZO subjected to 5 and 50 stretching cycles: (a) cell efficiency Eff.; (b) current density J_sc_; (c) electroluminescence images of cells after 50 stretching cycles, with and without Ag mesh.

Figure 7 presents a second experiment series where the number of stretching cycles was increased to 50 with an identical constant 2% strain ratio. The cells performance again degrades further when increasing the number of cycles from 5 to 50. For both 50 nm and 900 nm AZO thicknesses, the cells degradation is slowed down by the presence of the Ag mesh, as shown in Figure 7(a). After 50 cycles, the efficiency drops down to 58% efficiency instead of 40% for 50 nm AZO thickness, and to 53% instead of 26% for 900 nm thickness. These efficiency losses are mainly due to current density decreases (Figure 7(b)), while V_oc_ and fill factor experience a similar decrease in each of the configurations but independently of the presence of the Ag mesh. Additionally, electroluminescence images in Figure 7(c) also imply the existence of crack induced dead areas in cells only with AZO as TCEs, while cells with hybrid TCEs show comparatively better homogeneity in collecting charge carriers. This demonstrates that the hybrid TCEs composed of AZO and Ag mesh can indeed improve the cell durability under mechanical stress.

## 4. Conclusions

Conductive Ag fiber meshes were fabricated by draw-spinning method. After UV illumination and millisecond flash lamp annealing, a sheet resistance below 20 Ω/sq and an optical transmittance above 85% in the visible light spectra was achieved. The Ag fiber meshes were applied onto flexible CIGS solar cells terminated with sputtered AZO. By testing various thicknesses for AZO from 0 to 900 nm the following conclusions could be made:

1. Ag meshes alone are not suitable as TCE for flexible CIGS solar cells. Most photogenerated charge carriers get lost before reaching the point contacts to the Ag mesh and the resulting short current density is very low.
2. Hybrid TCE electrodes can enhance the charge carrier extraction in the long wavelength range and improve the fill factor when combined with thin (50 nm) AZO layers. No improvements in PV parameters were observed for combinations with thicker 200 or 900 nm AZO layers.
3. The cell stability under mechanical stress is improved by the presence of an Ag mesh. It is suggested that the Ag mesh helps to maintain a low series resistance of the hybrid TCE by bridging cracks that are formed in the AZO layer during mechanical stress tests.

In summary, Ag mesh has to be used in combination with a TCO layer for obtaining efficient CIGS solar cells. Even if the Ag mesh reduces light transmittance due to shadowing effect, a positive effect of the improved mechanical stability of the AZO/Ag mesh hybrid electrodes could be considered for larger-area flexible PV modules, whenever frequent module flexing is desired.
